# FGF-9 accelerates epithelial invagination for ectodermal organogenesis in real time bioengineered organ manipulation

**DOI:** 10.1186/1478-811X-10-34

**Published:** 2012-11-23

**Authors:** Yun-Yuan Tai, Rung-Shu Chen, Yi Lin, Thai-Yen Ling, Min-Huey Chen

**Affiliations:** 1 Graduate Institute of Clinical Dentistry, School of Dentistry, National Taiwan University, Taipei, 10002, Taiwan; 2 Graduate Institute of Pharmacology, College of Medicine, National Taiwan University, Taipei, 10002, Taiwan; 3 Dental Department, National Taiwan University Hospital, Taipei, 10002, Taiwan; 4 Research Center of Developmental Biology and Regenerative Medicine, National Taiwan University, Taipei, 10002, Taiwan

**Keywords:** FGF-9, Epithelial invagination, Ectodermal organogenesis, Combined bioengineered organ-ECIS model, FGF-BMP balancing system

## Abstract

**Background:**

Epithelial invagination is important for initiation of ectodermal organogenesis. Although many factors regulate ectodermal organogenesis, there is not any report about their functions in real-time study. Electric cell-substrate impedance sensing (ECIS), a non-invasive, real-time surveillance system, had been used to detect changes in organ cell layer thickness through quantitative monitoring of the impedance of a cell-to-microelectrode interface over time. It was shown to be a good method for identifying significant real-time changes of cells. The purpose of this study is to establish a combined bioengineered organ-ECIS model for investigating the real time effects of fibroblast growth factor-9 (FGF-9) on epithelial invagination in bioengineered ectodermal organs. We dissected epithelial and mesenchymal cells from stage E14.5 murine molar tooth germs and identified the real-time effects of FGF-9 on epithelial-mesenchymal interactions using this combined bioengineered organ-ECIS model.

**Results:**

Measurement of bioengineered ectodermal organ thickness showed that Fibroblast growth factor-9 (FGF-9) accelerates epithelial invagination in reaggregated mesenchymal cell layer within 3 days. Gene expression analysis revealed that FGF-9 stimulates and sustains early *Ameloblastin* and *Amelogenin* expression during odontogenesis.

**Conclusions:**

This is the first real-time study to show that, FGF-9 plays an important role in epithelial invagination and initiates ectodermal organogenesis. Based on these findings, we suggest FGF-9 can be applied for further study in ectodermal organ regeneration, and we also proposed that the ‘FGF-BMP balancing system’ is important for manipulating the morphogenesis of ectodermal organs. The combined bioengineered organ-ECIS model is a promising method for ectodermal organ engineering and regeneration research.

## Background

Ectodermal organogenesis involves the development of many ectodermal organs including hair, feathers, scales, teeth, beaks, nails, horns and several eccrine glands. Despite diverse forms and functions, ectodermal organs exhibit common developmental features. Originating from adjacent layers of epithelial (ectodermal) and mesenchymal (mesodermal or neural crest) tissues, the first visible sign of most ectodermal organs is a local epithelial thickening termed an ectodermal placode. Next, a condensation of mesenchymal cells, or papilla, forms under the placode. The ectodermal placode then buds into or out of the papilla in a process called epithelial invagination. Subsequent morphogenesis drives further development of the epithelial and mesenchymal components and is associated with epithelial folding and branching. This ultimately determines the final shape and size of ectodermal organs; however, it is epithelial invagination that regulates when and how ectodermal organs develop, a fact with important implications for future organ engineering and regeneration [for review see [1]].

Although many factors regulate ectodermal organogenesis, observing their functions in real-time is difficult. The complex developmental progress at different time points involves sophisticated spatial and temporal regulation of growth factor superfamilies including the Bone morphogenetic protein (BMP), Fibroblast growth factor (FGF), Tumour necrosis factor (TNF), Sonic hedgehog (Shh) and Wnt superfamilies [for review see [2-4]]. Hence, future study requires a more accurate model. The multipotency of embryonic stem cells makes creation of a non-invasive, non-inductive environment for the observation of specific growth factor functions within ectodermal organ development challenging. Ectodermal organs are greatly affected by their *in vivo* environment and its inherent regulating factors.

Bioengineered ectodermal organs can be developed from murine molar tooth germs by dissociating the epithelial and mesenchymal layers into single cells, then reaggregating them into a new organ (regenerated tooth germ). Previous studies have shown that these separated cells, once reaggregated and with the correct compartment relationship, can develop into complete, regenerated organs in vitro and *in vivo* [for review see [5-9]]. This bioengineered organ germ method [10] could facilitate regeneration of other ectodermal organs, even new engineered organs, which could be transplanted and function normally within humans [11]. However, because of the number of growth factors involved, methods for manipulation of ectodermal organogenesis in vitro and *in vivo* remain unelucidated.

Many growth factors are expressed during ectodermal organogenesis [12,13] and it is logical to study their specific roles during organ regeneration. Studies have shown that FGF-8 and FGF-9 are expressed within the murine molar epithelium: FGF-8 has a specific role in multicuspid odontogenesis [13,14]; therefore, in this study, we investigated the role of FGF-9, also present within the oral epithelium of the first branchial arch at stage E10. FGF-9 expression is restricted to the dental epithelium until epithelial budding at E11. Although not expressed in the mesenchyme, FGF-9 appears in the cap-stage enamel knot of the E13–E15 mouse embryo, spreading within the inner enamel epithelium until E18 [14]. This suggests that FGF-9 is an important factor during the initiation of tooth germ development. However, tissue slicing and staining cannot provide a real-time model for further investigation and, as FGF-9 is critical to embryonic development, a conventional transgenic approach could cause early defects, or even embryonic demise, interfering with in-depth study.

Electric cell-substrate impedance sensing (ECIS) is a non-invasive, real-time surveillance system used to detect changes in organ cell layer thickness through quantitative monitoring of the impedance of a cell-to-microelectrode interface over time [15]. It is a real-time, label-free, impedance-based method to study the activities of cells including morphological changes, locomotion, and other behaviors of cells directed by the cytoskeletons. When cells were added to the ECIS Arrays and attached to the electrodes, they play the roles as insulators and increased the impedance. As cells grow and cover the electrodes, the current is impeded in related with cell number, cell morphology, and cell attachment. When cells are stimulated, cell function and cell morphology will be changed and the impedance of cell-to-microelectrode will be changed. The data generated is impedance versus time. The high frequency impedance is caused by the increasing of cell numbers, whereas the low frequency impedance indicates the increasing of spaces between the cells. Previous study had reported that ECIS can be used to identify significant, real-time changes in organ layers caused by the invasive activities of cultured metastatic cells [16]. Because epithelial invagination is also related to the invasive activities of epithelial cells into mesenchymal cells, which indicated that ECIS would be a good method for detecting epithelial invagination in ectodermal organogenesis. The purpose of this study is to establish a combined bioengineered organ-ECIS model for investigating the real time effects of fibroblast growth factor-9 (FGF-9) on epithelial invagination in bioengineered ectodermal organs.

**Figure 1:**
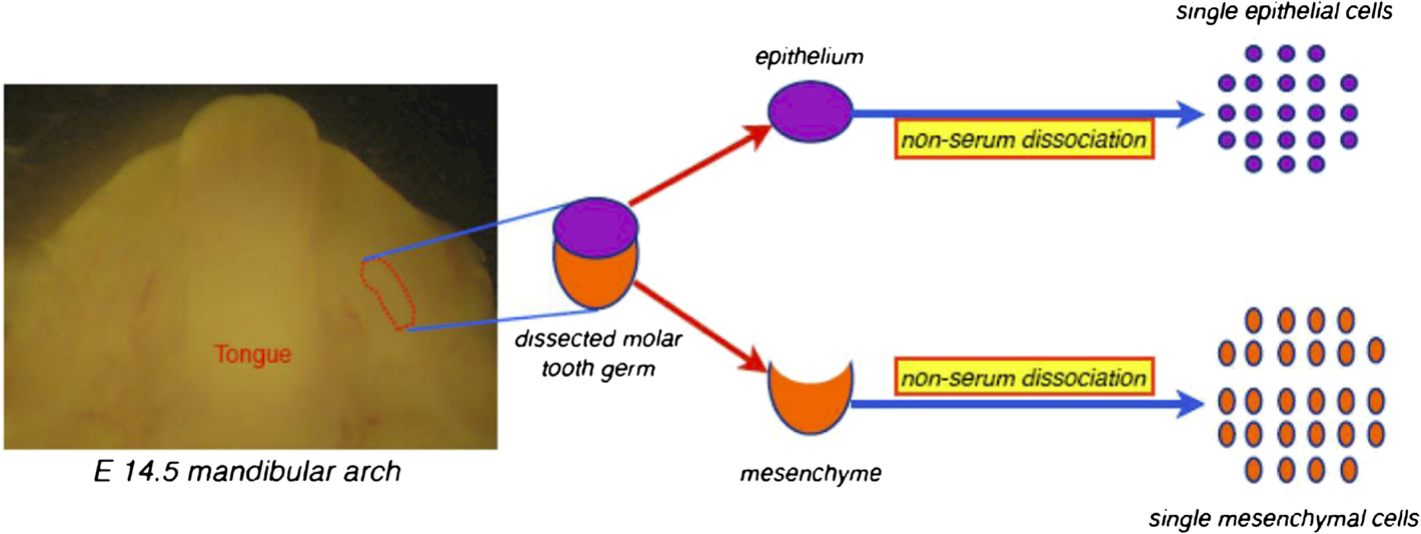
**Tooth germ dissection and dissociation.** First molar tooth germ dissection from the mandibular arch of an E14.5 mouse embryo and its dissociation into single cells using the non-serum protocol.

**Figure 2:**
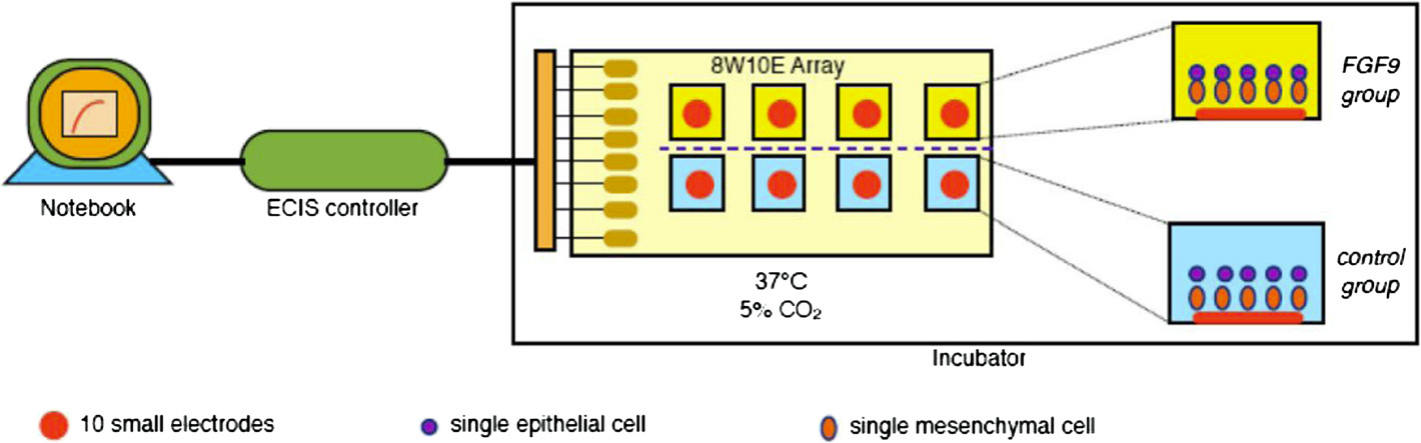
The combined bioengineered organ-ECIS model for analysis of epithelial invagination.

In this study, we aim to establish a non-invasive, real time model to investigate whether FGF-9 can promote epithelial invagination for ectodermal organogenesis, and whether there are important gene expressions related with the development of tooth germ during this event. We used the non-serum stem cell-separating method [17,18] to obtain purified, non-inductive, single epithelial and mesenchymal cells, dissected from the first molar tooth germs of stage E14.5 (equal to cap stage of tooth germ in development) imprinting control region (ICR) mouse embryos (Figure 1). These cells were reaggregated for the epithelial invasion test in an ECIS Z8 system (Applied Biophysics, Troy, NY, USA) and sustained with FGF-9 in the experimental group. This bioengineered organ-ECIS model provides a non-invasive, controlled environment for the real-time investigation of specific factors (Figure 2), avoiding mutagenesis and tissue slicing and staining. Gene expression assays performed at multiple time points will illuminate the mechanisms underlying ectodermal organogenesis.

## Results

### FGF-9 promotes tooth germ development

In preliminary experiments, we used FGF-9 cultured with the dissociated mesenchymal cells and the dissected lower arches from the E14.5 ICR embryos. Preliminary results showed that FGF-9 can induce early, significant *Ameloblastin*, *Amelogenin* and *Osteocalcin* expression in mesenchymal cells cultured with FGF-9 (Additional file 1). These findings were concurrent with the gene expression of mesenchymal origin cells, such as odontoblasts, during the early stage of tooth development [19,20]. In addition, FGF-9 can induce early expression of the *homeobox* protein *Msx* genes *Msx-1* in mesenchymal cells (Additional file 1), which is consistent with previous research [14]. The experimental group also exhibited earlier epithelial differentiation in molar tooth germs at 1 and 10 days in vitro (x 2 and 3). This indicated that the development of tooth germ was promoted by FGF-9, which inspired us to detect the role of FGF-9 in epithelial invagination.

For optimizing the effects of FGF-9 in promoting cell proliferation, cell proliferation rate with different concentrations of FGF-9 were compared by MTT assay. In this assay, yellow MTT (3-(4,5-Dimethylthiazol-2-yl)-2,5-diphenyltetrazolium bromide, a tetrazole) is reduced to purple formazan in the mitochondria of living cells. The absorbance of this colored solution can be quantified by measuring at a certain wavelength (usually between 500 and 600 nm) by a spectrophotometer. The absorption max is dependent on the solvent employed. This reduction takes place only when mitochondrial reductase enzymes are active, and therefore conversion can be directly related to the number of viable (living) cells. The rate of tetrazolium reduction is proportional to the rate of cell proliferation.

**Figure 3:**
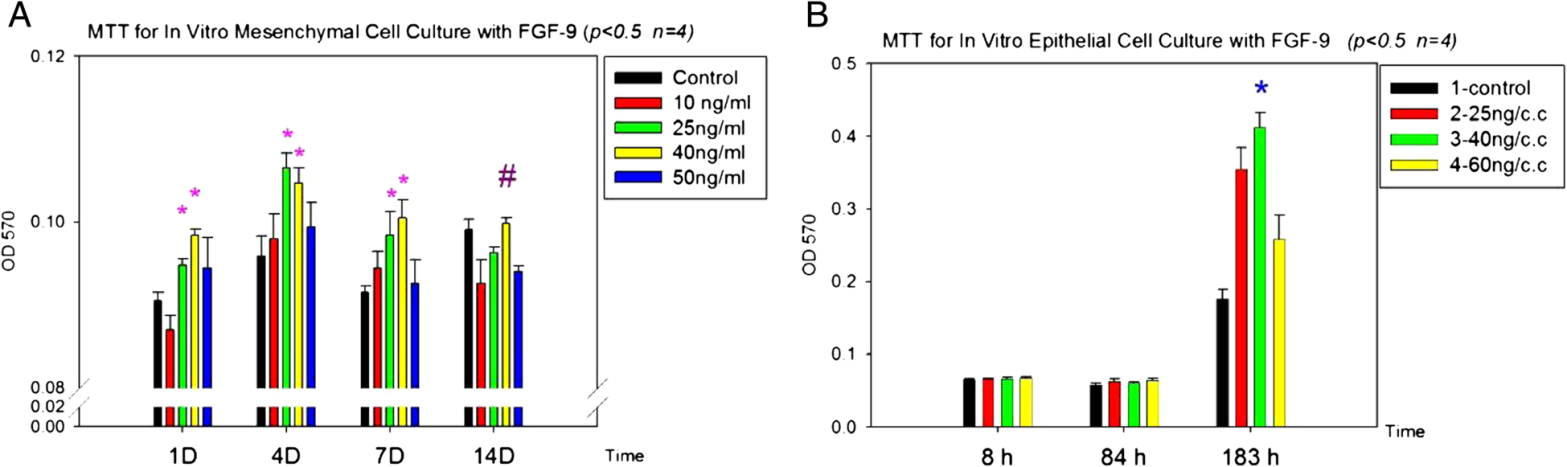
**MTT assay.** MTT assay for the in vitro culture of dissociated mesenchymal and epithelial cells to determine the optimal Fibroblast growth factor-9 (FGF-9) concentration. (**A**) MTT data for mesenchymal cells (*n = 4*, *p <* 0.5): FGF-9 concentrations of 25 and 40 ng/ml were both suitable for cell culture in the first 7 days. # : 40 ng/ml FGF-9 outperformed 25 ng/ml after 14 d of culture, (**B**) MTT for epithelial cells (*n = 4*, *p* < 0.5): 40 ng/ml is optimal for epithelial cell culture within the first 183 h (> 7 days). Thus, we chose 40 ng/ml FGF-9 for further experimentations.

Subsequently, we found FGF-9 with 40 ng/ml concentration was the best for promoting cell proliferation in both dissociated epithelial cells and mesenchymal cells (Figure 3).

MTT data suggested that FGF-9 facilitates early mesenchymal cell proliferation (Figure 3A). MTT data also suggested that FGF-9 can stimulate epithelial cell proliferation at 183 h (Figure 3B). Therefore, we used the ECIS invasion test [16] to identify the role of FGF-9.

### ECIS impedance change reveals FGF-9 stimulates accelerated epithelial invagination

**Figure 4:**
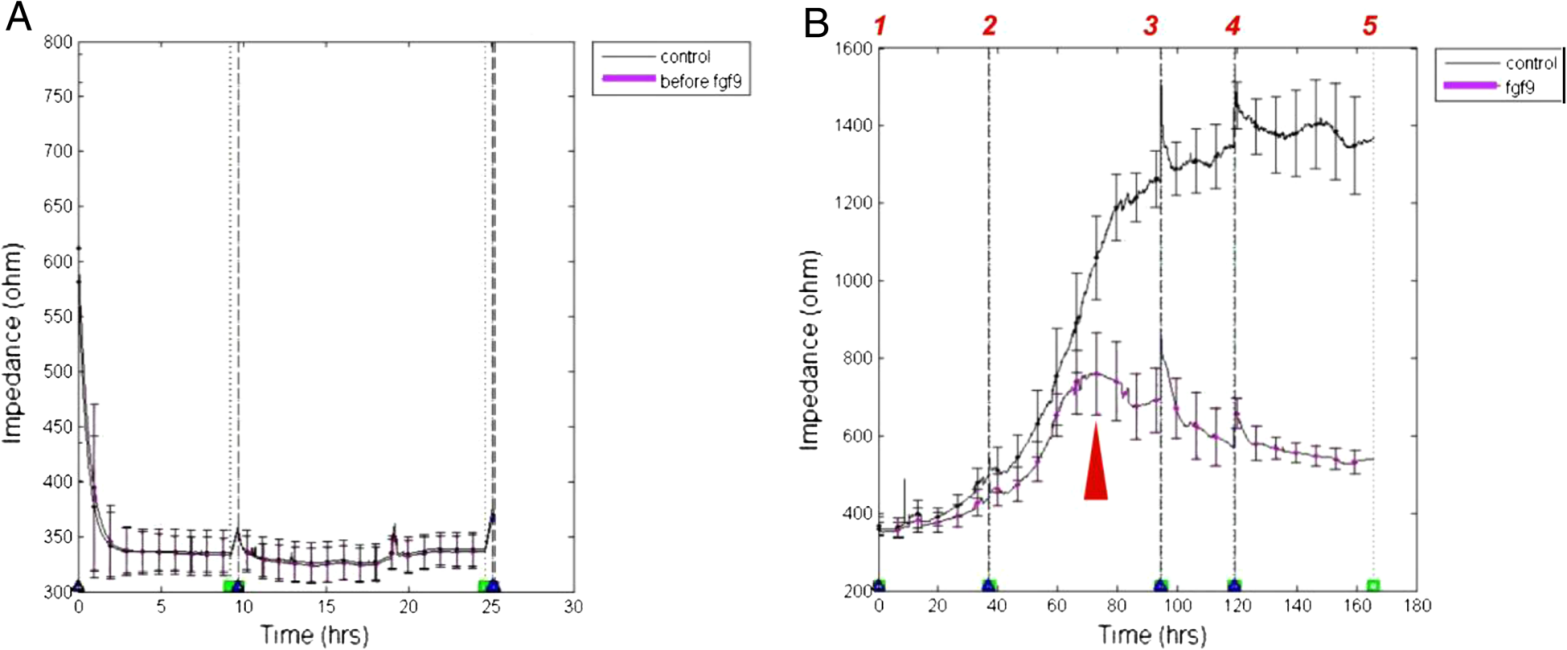
**ECIS Assay.** We chose the ECIS Z8 system, with four wells chosen as the control group, and the other four wells as the FGF-9 group. The ECIS parameters include a frequency of 15,000 Hz and impedance data were recorded every 90 s. The black line represents the control group and the pink line represents the FGF-9 group. The vertical (dash) line represents the time of medium change. (**A**) Before adding epithelial cells and FGF-9 (40 ng/ml), it was necessary to allow mesenchymal cells to reaggregate as a stable layer in each well of the ECIS kit. We then added 2 × 10^5^ mesenchymal cells to each well. Impedance data showed no difference between the two groups in the first 25 h, indicating no significant proliferation or death of mesenchymal cells. (**B**) The invasion test began after the addition of epithelial cells and FGF-9 (40 ng/ml). We added 2 × 10^5^ epithelial cells to each well of the 8W10E kit. We added ordinary fresh medium to the control group (black line) and fresh medium with FGF-9 (40 ng/ml) to the FGF-9 group (pink line). The vertical (Dash) line:*1*. Start invasion. *2–4*. Change half medium in each well. *5*. Stop observation at 163 h. Red arrow: significant time point (approximately 72–74 h) of impedance change in the FGF-9 group, when epithelial invagination starts to result in a thinner layer of bioengineered organ.

After dissecting the first molar tooth germ from E14.5 ICR embryos, we separated the epithelial and mesenchymal layers, obtaining single cells through the non-serum dissociation method [17,18]. We added 2 × 10^5^ mesenchymal cells to each well of the ECIS 8W10E kit (Applied Biophysics), waited 25 h for settling and reaggregation and obtained stable impedance (Figure 4A). We investigated epithelial cell invasion by adding 2 × 10^5^ epithelial cells to each well (Figure 2). The FGF-9 concentration was maintained at 40 ng/ml in the four experimental wells to detect enhanced epithelial cell layer invagination into the established mesenchymal layer (*1*, Figure 4B). Early impedance of the FGF-9 group was always lower than the control group, although not significantly (Figure 4B). This suggests initial epithelial invagination proceeds from the very first time point.

After 72 h (red arrow, Figure 4B), the FGF-9 group exhibited great variation in impedance data (pink line, Figure 4B), suggestive of significantly thinner bioengineered organ layers than that of the control group. According to the MTT data, FGF-9 does not jeopardize tooth germ cell activity (Figure 3). Therefore, accelerated epithelial invagination occurred before this time point. This may explain significant FGF-9 expression in the epithelium [14] and earlier differentiation of tooth germ we observed in our preliminary in vitro organ culture experiments (Additional file 2 and 3).

Although changing half the medium could have affected impedance data for a short time, each group tended to persist in its own manner (*2–4*, Figure 4B). After a 163-h observation period, there was no great change in the control group impedance data, which remained stable (black line, Figure 4B). We inferred that the well of the 8W10E kit did not provide enough space for further cell proliferation, making it pointless to continue the ECIS study beyond this time point (*5*, Figure 4B). The impedance data showed that the bioengineered organ increased in thickness approximately fourfold within 7 days. During this period, electrical resistance increased from <400 Ω to >1400 Ω (Figure 4B). Although well size was limited during organ development, we attempted to improve the culture environment by changing the medium in the last 80 h. No further changes in impedance occurred in either the control or FGF-9 groups after this (Figure 4B), indicating that only epithelial thickening occurred.

### FGF-9 accelerates and sustains *Ameloblastin* and *Amelogenin* expressions

ECIS data revealed significant electric impedance changes during the 60–84-h period (*2–3*, Figure 4B). We performed mRNA detection at different time points to determine the factors underlying ectodermal organogenesis. Using the same culture protocol as for previous ECIS surveillance, we examined expression of two genes critical to normal tooth germ development, *Ameloblastin* and *Amelogenin*[21,22]. *β-actin* served as an internal control for real-time 7900HT PCR.

**Figure 5:**
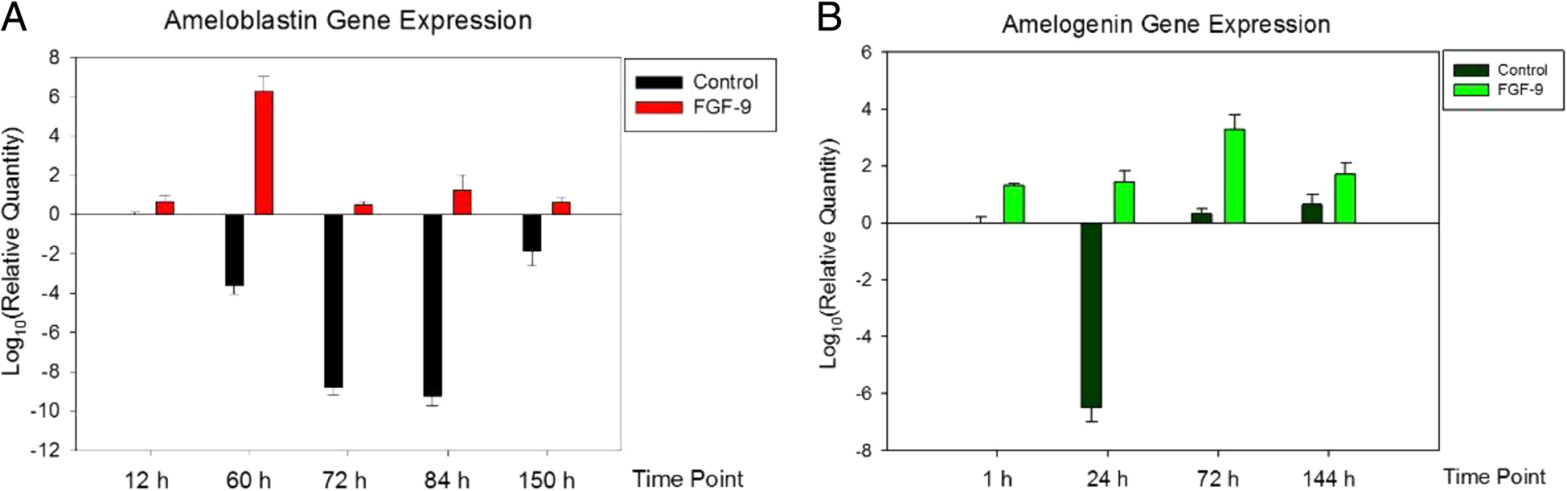
**Real-time PCR measurement of *Ameloblastin *and *Amelogenin *gene expression.** (**A**) Real-time PCR *Ameloblastin* expression at different time points in the bioengineered organ culture. *Ameloblastin* expression peaked at 60 h. *Ameloblastin* expression was significantly higher at 72, 84 and 150 h in the FGF-9 group compared with the control group. (**B**) Real-time PCR *Amelogenin* expression at different time points of the bioengineered organ culture. *Amelogenin* expression was significantly higher in the FGF-9 group compared with the control group at 1 h and peaked at 72 h, coinciding with a significant change in ECIS assay (Figure 4B) and due to epithelial invagination. These two graphs show that the peak *Ameloblastin* expression at 60 h is followed by a peak *Amelogenin* expression at 72 h, in agreement with previous research [21]. Significantly higher *Amelogenin* expression also coincides with early lower impedance data (Figure 4B) and earlier differentiation of tooth germ evident in early organ culture (Additional file 2). These results indicate that FGF-9 can initiate early phenotypic and morphological characteristics of ameloblasts within the epithelium, which coincides with epithelial invagination evident from the ECIS assay (Figure 4B). After invagination, the FGF-9 group still exhibits higher *Ameloblastin* and *Amelogenin* expressions than the control group. Because of the clear differences, no mark is necessary to specify the results of the FGF-9 group.

Because *Ameloblastin* expression precedes *Amelogenin* expression [21], we established different time schedules for their analysis: we monitored *Ameloblastin* expression at 12, 60, 72, 84 and 150 h (Figure 5A), whereas we monitored *Amelogenin* expression at 24, 72 and 144 h (Figure 5B). Owing to very low *Amelogenin* expression, we also detected *Amelogenin* expression at 1 h (Figure 5B) to determine why early impedance is lower in the FGF-9 than the control group. *Ameloblastin* was significantly elevated by FGF-9 at 12 h (Figure 5A) relative to the control group. Peak *Ameloblastin* expression was evident at 60 h in the FGF-9 group (Figure 5A). This suggests that FGF-9 accelerates early *Ameloblastin* expression and induces early accelerated epithelial invagination within 72–74 h. FGF-9 also accelerated and sustained *Ameloblastin* expression during the observation period (Figure 5A). Even at 150 h, the FGF-9 group exhibited significantly higher *Ameloblastin* expression. Combined with the comparatively lower impedance data of the FGF-9 group (Figure 4B), the evidence suggests that addition of FGF-9 induces early *Ameloblastin* expression, some early phenotypic characteristics of ameloblasts existed within the epithelial layer and accelerated epithelial invagination.

Trace *Amelogenin* expression was evident in the FGF-9 group after 1 h (Figure 5B), explaining the early lower impedance of the FGF-9 group. Consequently, early morphological characteristics of ameloblasts occurred, concurring with in vitro organ culture results (Additional file 2). Although *Amelogenin* expression was very weak, it was higher in the FGF-9 group than in the control group, peaking at 72 h (Figure 5B) following peak *Ameloblastin* expression at 60 h (Figure 5A) [21,22]. FGF-9 sustained greater *Amelogenin* expression than that exhibited by the control group, even after 144 h (Figure 5B). These results provide solid evidence that FGF-9 accelerates epithelial invagination and early phenotypic and morphological characteristics of ameloblast existed within the epithelium.

## Discussion

Because of the multipotency of embryonic stem cells, it is difficult to create a non-invasive and non-inductive environment for observing how a specific growth factor works within ectodermal organ development. Ectodermal organs are always affected by the surrounding *in vivo* environment and many types of regulating factors, that might be upregulated or downregulated, or work as feedback balancing systems.

We employed a combined bioengineered organ-ECIS model using tooth germ cells (Figure 2), observing their real-time development within a non-invasive, ECIS surveillance system. The method also facilitated assessment of the effect of FGF-9 on epithelial invagination over time (Figure 4B) without tissue slicing or staining [16].

FGF-9 accelerates epithelial invagination; however, FGF-9 is only expressed in the epithelium, not in the mesenchyme. FGF-9 is known to function in several reciprocal epithelial–mesenchymal interactions: stimulating proliferation of mesenchymal cells (Figure 3A); inducing early mesenchymal expression of the crucial odontogenic genes *Msx-1*, *Ameloblastin* and *Amelogenin* (Additional file 1) [14]; and accelerating and sustaining early Ameloblastin and Amelogenin secretion within the tooth germ (Figure 5). Thus, FGF-9 may drive early differentiation of the epithelial cell layer to express phenotypic and morphological characteristics of ameloblasts (Additional file 2 and 3), cause proliferation of the epithelium for invagination (Figure 3B) and promote ectodermal organogenesis. This could explain why FGF-9 is restricted to the epithelium and persists there [14]: to initiate epithelial budding. Our findings also suggest that FGF-9 could play an important role in ectodermal organ regeneration and tissue engineering.

MTT data suggested that FGF-9 facilitates early mesenchymal cell proliferation (Figure 3A), possibly also increasing endogenous FGF-10 in mesenchymal cells during proliferation. FGF-10 is known to stimulate proliferation in epithelial cells [23], potentially creating larger tooth germs. MTT data also suggested that FGF-9 can stimulate epithelial cell proliferation at 183 h (Figure 3B). Resent research indicates that a TGFβ-FGF9-PITX2 signaling cascade regulates cranial neural crest cell proliferation in palatal mesenchyme during palate formation [24]. It seems FGF-9 can regulate the proliferation and integration within mesenchymal cells in a more specific route, which also control the interaction between ectodermal epithelium and mesenchyme. Previous research [5,25] demonstrated using a bioengineered organ culture system similar to ours that epithelial invagination occurred after 1 week of in vitro culture: if dissociated epithelial cells recombined with intact dissected mesenchymal layers directly, new tooth germs formed faster than that with reaggregated mesenchymal cell layers [5,25]. Our FGF-9 group exhibited faster mesenchymal cell integration in the first 3–4 days than previous studies (Figure 4B) [5,6,25]. These results suggest that FGF-9 functions in assisting mesenchymal cells to closely re-integrated, similar to a tissue layer. This might also explain how FGF-9 accelerates epithelial invagination in murine odontogenesis: by facilitating cross-talk and reunion between mesenchymal cells and stimulating epithelial cell layer differentiation, creating an ameloblast layer. Thus, FGF-9 may play a significant role in future stem cell organ regeneration.

**Figure 6:**
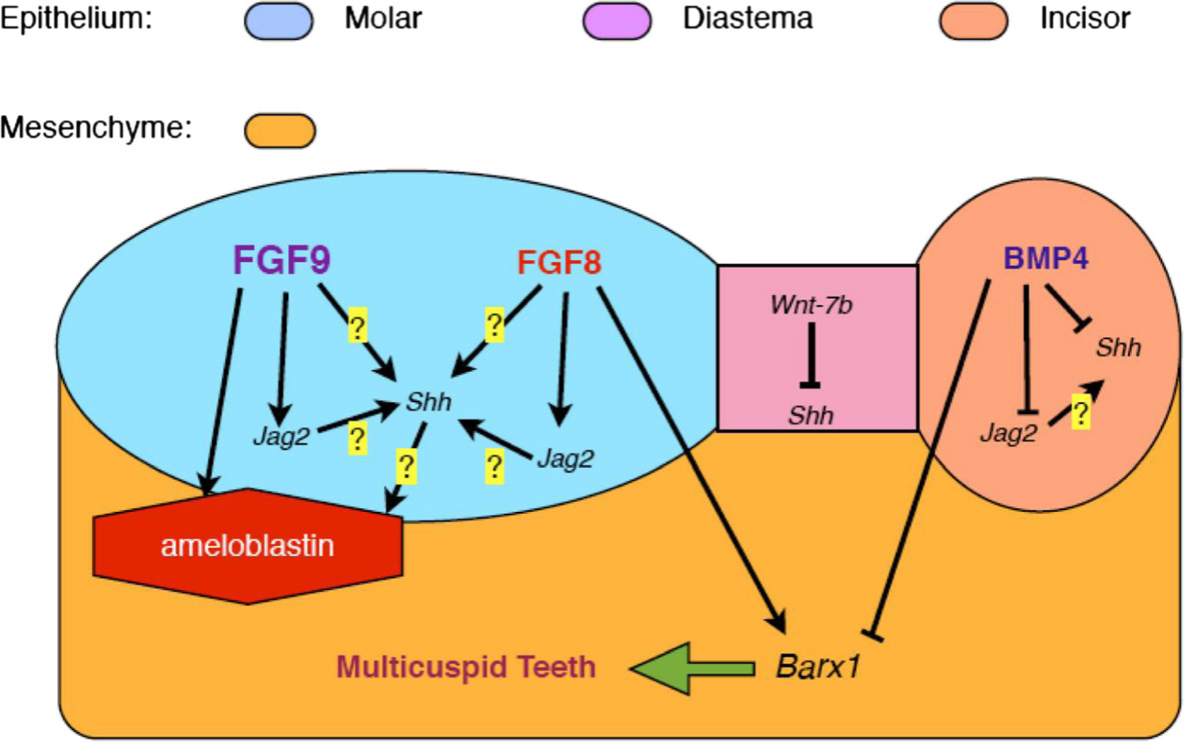
**Proposed mechanism of FGF-BMP balancing system.** In this study, FGF-9 upregulates *Ameloblastin* and *Amelogenin*, and speeds up epithelial invagination. No direct relationship between *Sonic hedgehog (Shh)*, *Jagged (Jag2)* and *Ameloblastin* has been elucidated until now. Other genes (such as *Paired box 9*) were found to support antagonistic interactions between FGFs and BMPs [32] but are not shown in this graph. We propose an ‘FGF-BMP balancing system’ which manipulates the morphogenesis of ectodermal organs. “→” is the upregulate pathway proved by previous researches and our study. “⊥” is the downregulated pathway proved by previous research. “?” is the suggested pathway need to be investigated further.

Some authors have suggested that *Wnt-7b* restricts dental organ development to within the maxillary and mandibular arches [26]. Ectopic epithelial *Wnt-7b* expression, achieved through murine retrovirus infection, downregulates Shh expression within dental organs, completely abrogating their development; however, subsequent local Shh overexpression through beads revives tooth germ development [26]. This suggests that FGF-9 counterbalances *Wnt-7b* through the Shh pathway. Previous evidence [27] demonstrated that suppressing Shh created a similar phenotype to BMP4 overexpression in the feather model. We propose that FGF-9 plays a significant role in modulating the BMP-Shh ‘signalling module’ regulating skin appendage morphogenesis [27-29] by accelerating epithelial invagination. Our preliminary results show that FGF-9 upregulates *Msx-1* expression within a mesenchymal cell culture (Additional file 1). Previous studies [30] showed that FGFs and BMP4 induced both *Msx1*-independent and -dependent signalling pathways in early odontogenesis. These results suggest that FGFs and BMPs might influence the same regulatory systems functioning in multiple developmental areas and time points. A significant, proximal–distal pattern exists within the lower jaw epithelium of the mouse embryo [13]: FGF-8 and FGF-9 are expressed in the lateral molar field whereas BMP4 is expressed over the medial incisor area. This specific pattern suggests that FGF-8 and FGF-9 correlate with multicuspid odontogenesis whereas BMP4 correlates with unicuspid odontogenesis. Recent research [31] showed that both BMPs and FGFs target the Notch signalling pathway through the *Jagged 2* (*Jag2*) gene, regulating tooth morphogenesis and cytodifferentiation. However, *Jag2* expression is downregulated by BMPs but upregulated by FGFs within the dental epithelium. This suggests a specific, counterbalanced relationship exists between FGFs and BMPs. If BMP overexpression suppresses Shh expression, then FGF overexpression may promote Shh expression, accelerating epithelial invagination. We propose an ‘FGF-BMP balancing system’ (Figure 6), [32] which determines the cusp number in odontogenesis and may similarly regulate feather or skin appendage morphogenesis. These topics are interesting issues for further discussion and require future research.

## Conclusions

The bioengineered organ-ECIS model provides a novel understanding of FGF-9 function in ectodermal organogenesis and insights into methods of manipulating the speed of ectodermal organ regeneration. These results directly support the role of FGF-9 in epithelial invagination. Ours is the first study to show that FGF-9 is, in addition to BMP-Shh signalling [26-31,33], a major regulator of epithelial invagination during the initiation of ectodermal organogenesis, with future applications in ectodermal organ regeneration and tissue engineering.

## Methods

### Animals

Stage E14.5 pregnant female ICR mice were purchased from BioLASCO Taiwan Co., Ltd. (Taipei, Taiwan). Mouse care and handling procedures, and experimental protocols were approved by the National Taiwan University of Science Animal Care and Use Committee, and conducted in accordance with Directive 86/609/EEC for animal experiments.

### First molar tooth germ dissection and dissociation into single cells through the non-serum protocol

Under a stereomicroscope (Leica MZ7.5; Leica Microsystems, Wetzlar, Germany), we microsurgically dissected first molar tooth germs from the mandibular arch of stage E14.5 (equal to cap stage of tooth germ in development) embryos. After soaking the dissected tooth germs in dispase (1.2–1.5 U/ml; Invitrogen, Carlsbad, CA, USA) for 8–10 min at 4°C, we separated the epithelial and mesenchymal layers using a tungsten needle. The separated layers were stored immediately in 400 μl Spinner modified Ca^2+^-free minimal essential medium (SMEM; Sigma Chemical Co., St. Louis, MO, USA) at 4°C in a 1.5 ml microcentrifuge tube (Eppendorf, Hamburg, Germany).

We prepared 5 ml non-serum dissociation medium (Ling et al., 2006; Huang et al., 2009) by adding 0.01 g protease XIV (Sigma) and 0.02 g DNase (Bioshop Canada Inc., Burlington, CA, USA) to 5 ml SMEM and filtering the solution with a 0.22 μm filter (Millex Syringe Filter Unit; EMD Millipore Corporation, Billerica, MA, USA). We introduced 400 μl filtered non-serum dissociation medium into the stored cell layers. Dissociation proceeded for 12–14 h at 4°C. After gentle pipetting, we centrifuged the mixture twice at 1250 revolutions per minute (rpm) for 5 min (Kubota 2420 Centrifuge; Kubota Corp., Osaka, Japan). Discarding the supernatant, we added 1 ml Dulbecco’s modified Eagle’s medium (DMEM; Invitrogen), mixed and counted cell numbers (Figure 1).

### 3-(4,5-Dimethylthiazol-2-yl)-2,5-diphenyltetrazolium bromide assay

We mixed 3-(4,5-dimethylthiazol-2-yl)-2,5-diphenyltetrazolium bromide (MTT) crystals (Sigma) with 30–40 ml phosphate-buffered saline, creating a 5 mg/ml solution. We filtered this with a 0.22 mm filter (Millipore), covered it with aluminium foil and stored it at 4°C. Next we added 2×10^4^ cells/well epithelial and mesenchymal cells to a 96-well plate. We added 200 μl/well of a range of FGF-9 (PeproTech Inc., Rocky Hill, NJ, USA) concentrations—10, 25, 40 and 50 ng/ml—to mesenchymal cell cultures. After 1, 4, 7 and 14 days’ incubation (*n* = 3–4), we removed the medium, added 30 μl/well MTT solution in darkness, then incubated for 3 h at 37°C. We removed the MTT solution, added 50 μl/well dimethyl sulphoxide and agitated for 10–15 min until the purple crystals dissolved. We performed enzyme-linked immunosorbent assay at 570 nm optical density.

After acquisition of mesenchymal cell MTT data, we added 200 μl/well of a range of FGF-9 concentrations—25, 40 and 80 ng/ml-for epithelial cell culture, analysing at 1, 4, 7 and 14 days.

### Bioengineered organ culture via electric cell-substrate impedance sensing

Owing to the limited quantity of the primary epithelial and mesenchymal cells, we chose the ECIS Z8 (Applied Biophysics) as an appropriate system to investigate the real time invagination effect of the primary epithelial cells as soon as possible. We added 2×10^5^ mesenchymal cells to each well of the 8W10E ECIS kit (Applied Biophysics). We set the frequency to 15000 Hz, collecting impedance data every 90 seconds. We stopped ECIS after 25 h to allow the mesenchymal cells to settle. We then added 2×10^5^ epithelial cells in 400 μl/well medium to each well, maintaining the FGF-9 concentration of the experimental group at 40 ng/ml. We maintained the control group in 400 μl/well fresh DMEM. We restarted ECIS impedance recording, changing half the medium at 36, 96 and 120 h (Figure 2).

### 7900HT real-time polymerase chain reaction gene expression assay and statistical analysis

For *7900HT real-time PCR gene expression analysis*, we followed the ECIS assay protocol as described previously. We measured *Ameloblastin* expression at 12, 60, 72, 84 and 150 h and *Amelogenin* expression at 1, 24, 72 and 144 h. To replicate the ECIS 8W10E kit environment, we used 48-well plates for *Ameloblastin* and *Amelogenin* analysis. It was necessary to accommodate this mass and pool data from different time points using the real-time PCR software SDS 2.4 and RQ Manager 1.2.1 (Applied Biosystems, Foster City, CA, USA).

We purified RNA collected at different time points using an Rneasy® Mini Kit (Qiagen, Hilden, Germany) and PureLink® RNA Mini Kit (Invitrogen). We converted mRNA to cDNA using a High Capacity RNA-to-cDNA Kit (Invitrogen) and a T1 Plus Thermocycler (Montreal Biotech Inc., Dorval, PQ, Canada). We used the TaqMan® Gene Expression Assay for *Ameloblastin* (Mm00711644_g1; Invitrogen), *Amelogenin* (Mm00477486_m1; Invitrogen) and the internal control *β-actin* (Mm01205647_g1; Invitrogen) in the 7900HT Fast Real-Time PCR System (Invitrogen). After TaqMan® Gene Expression Master Mix (Invitrogen), we added the cDNA to the reaction plate and centrifuged the mixture (3500 rpm, 4°C, 5 seconds) to spin down the contents of each well. Then, we performed 7900HT PCR to determine relative gene expressions over time.

We marked at least four samples for each time point from the control and FGF-9 groups. We divided the cDNA from each sample into three wells to determine the expression of each gene in triplicate assay in a 96-well reaction plate. After 7900HT PCR, we pooled the data from SDS 2.4 into RQ Manager 1.2.1 to determine the average relative quantity of each sample, then used a base-10 logarithm to determine gene expressions. Next, we calculated the standard deviation of both groups at each time point and used the paired *t-*test for statistical analysis. We entered this data into SigmaPlot 10.0 (Systat Software Inc., San Jose, CA, USA) to construct gene expression plots for *Ameloblastin* and *Amelogenin* over time.

## Competing interests

We declare no conflict of interest in this work.

## Authors’ contributions

Y-YT, and M-HC prepared the research design; Y-YT, and YL performed research; T-YL contributed new reagents/analytic tools; Y-YT, R-SC, T-YL, and M-HC analyzed data; and Y-YT, T-YL, and M-HC wrote the paper. All authors read and approved the final manuscript.

## Supplementary Material

Additional file 1 **Preliminary experiment: FGF-9 upregulates *Ameloblastin *and *Amelogenin *in cultured mesenchymal cells.** Mesenchymal cells were cultured in vitro and FGF-9 (25 ng/ml) was added to the experimental group. FGF-9 significantly upregulated *Ameloblastin* and *Amelogenin* expression [19,20]. The homeobox protein *Msx genes Msx-1* and *Msx-2* (although expressed at low levels), and *Osteocalcin* were also upregulated after initial contact with FGF-9. *Msx-1* and *Msx-2* are critical to tooth germ development. Upregulation of *Msx-1* and weak expression of *Msx-2* evident in this study agree with previous research [14]. *Osteocalcin* was upregulated by FGF-9 in the first week and high expression was sustained compared with the control group, only diminishing temporarily at the 14^th^ day. These results highlight the importance of FGF-9 in ectodermal organogenesis. (A) Electrophoresis of *Ameloblastin, Amelogenin*, *Msx-1*, *Msx-2*, *Osteocalcin,* and *β-actin* expression. We used *β-actin* as internal control. (B) *Ameloblastin* expression of mesenchymal cell cultured with FGF-9 in vitro, (C) *Amelogenin* expression, (D) *Msx-1* expression, (E) *Msx-2* expression, and (F) *Osteocalcin* expression. (G) Internal control of the preliminary experiment: *β-actin* expression. *(n = 3, p < 0.5) CD: control group. FD: FGF-9 group.* Click here for file

Additional file 2 **Haematoxylin and eosin staining of ectodermal organ culture.** Day-1. *(n=4)* (A) and (B) control group Day 1. (C) and (D) FGF-9 group Day 1. Tooth germs cultured with FGF-9 demonstrated more differentiated than that in the control group. In this study, eight embryonic tooth germs were cultured, 4 for experimental group cultured with FGF-9, 4 for control group cultured without FGF-9. Some mineralized epithelium and morphological characteristics of ameloblasts were found in the experimental group with FGF-9, (C) and (D). (A) and (C): 200×; Bar = 12.5 μm. (B) and (D): 400×; Bar = 25 μm; *1: epithelium. 2: mesenchyme*. Click here for file

Additional file 3 **Haematoxyliin and eosin staining of ectodermal organ culture.** Day 10. *(n=4)* Eight embryonic tooth germs were cultured, 4 for experimental group cultured with FGF-9, 4 for control group cultured without FGF-9. (A) control group Day 10. Only simple epithelium and mesenchyme in the tooth germ was found. *1: epithelium. 2: mesenchyme.* (B) FGF-9 group Day 10. The FGF-9 group showed a more complex tooth germ structure after organ culture in vitro for 10 days. *3: some morphological characteristic of ameloblasts. 4: some morphological characteristic of odontoblasts. 5: mesenchyme.* Bar = 25 μm. Magnification 400×. Click here for file
