# Supplementary material for: FGF-9 accelerates epithelial invagination for ectodermal organogenesis in real time bioengineered organ manipulation
Source: Cell Commun Signal. 2012 Nov 23;10:34. doi: 10.1186/1478-811X-10-34 (PMC3515343; doi:10.1186/1478-811X-10-34)
Supplement: Additional file 1 — Preliminary experiment: FGF-9 upregulates Ameloblastin and Amelogenin in cultured mesenchymal cells. Mesenchymal cells were cultured in vitro and FGF-9 (25 ng/ml) was added to the experimental group. FGF-9 significantly upregulated Ameloblastin and Amelogenin expression [19,20]. The homeobox protein Msx genes Msx-1 and Msx-2 (although expressed at low levels), and Osteocalcin were also upregulated after initial contact with FGF-9. Msx-1 and Msx-2 are critical to tooth germ development. Upregulation of Msx-1 and weak expression of Msx-2 evident in this study agree with previous research [14]. Osteocalcin was upregulated by FGF-9 in the first week and high expression was sustained compared with the control group, only diminishing temporarily at the 14th day. These results highlight the importance of FGF-9 in ectodermal organogenesis. (A) Electrophoresis of Ameloblastin, Amelogenin, Msx-1, Msx-2, Osteocalcin, and β-actin expression. We used β-actin as internal control. (B) Ameloblastin expression of mesenchymal cell cultured with FGF-9 in vitro, (C) Amelogenin expression, (D) Msx-1 expression, (E) Msx-2 expression, and (F) Osteocalcin expression. (G) Internal control of the preliminary experiment: β-actin expression. (n = 3, p < 0.5) CD: control group. FD: FGF-9 group. [file 1478-811X-10-34-S1.doc]

**Additional file 1**

A


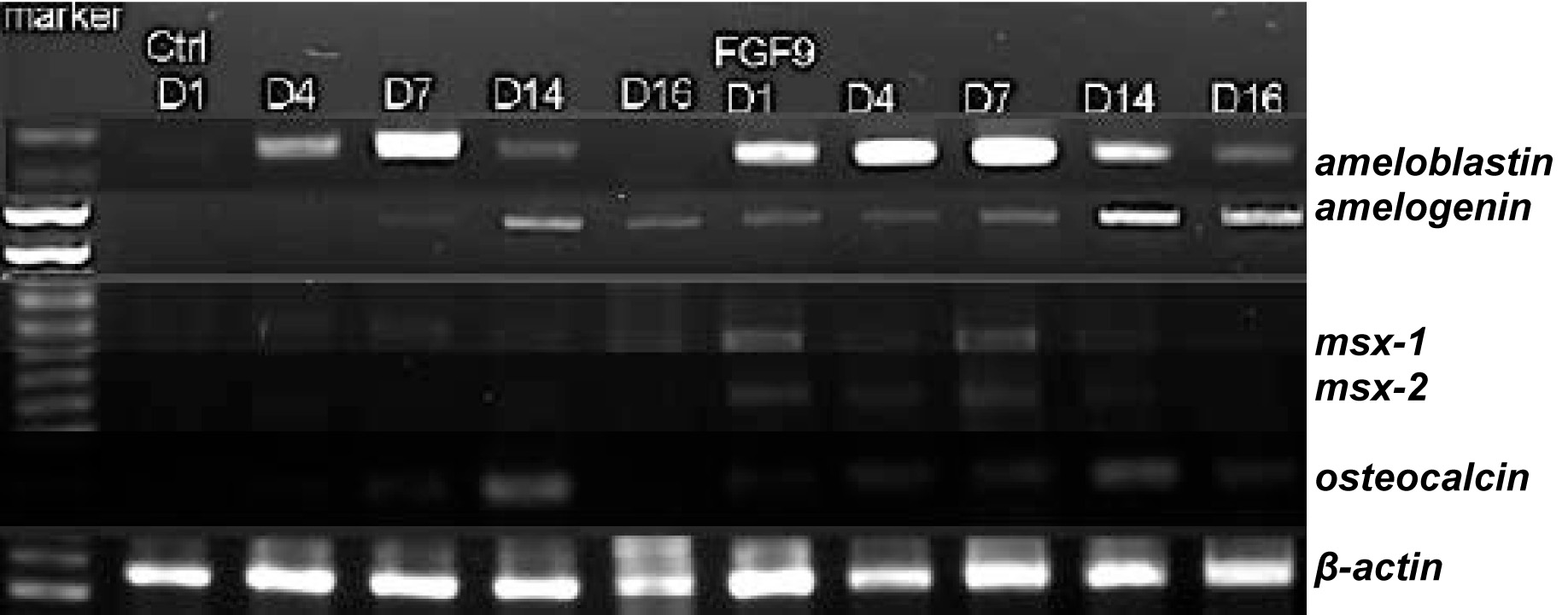


B C


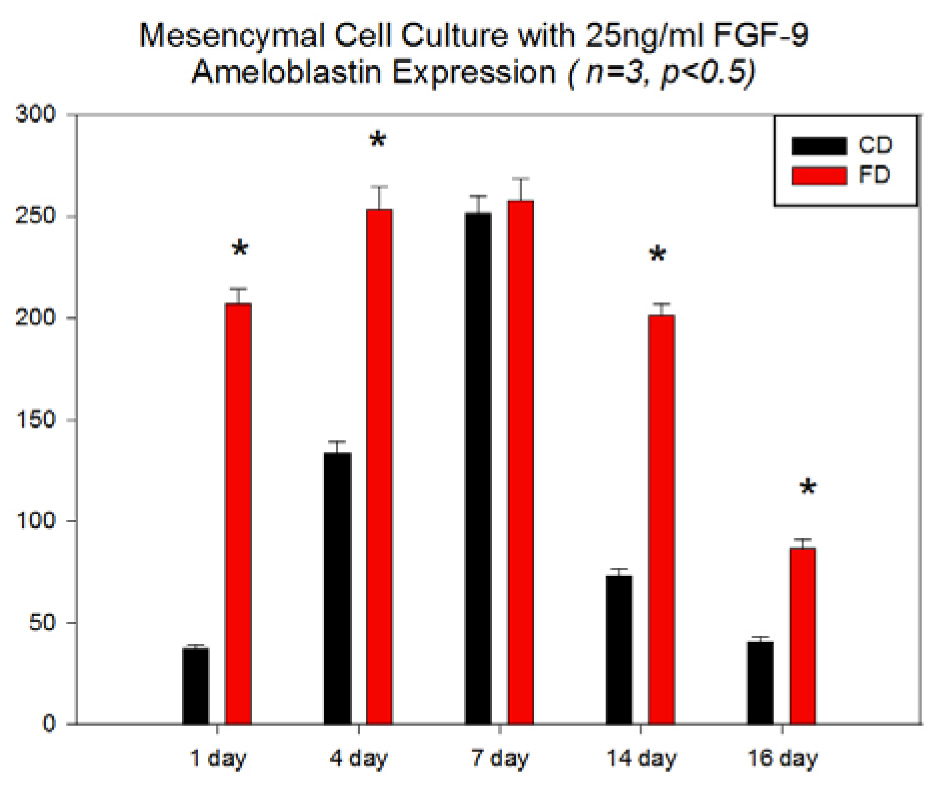

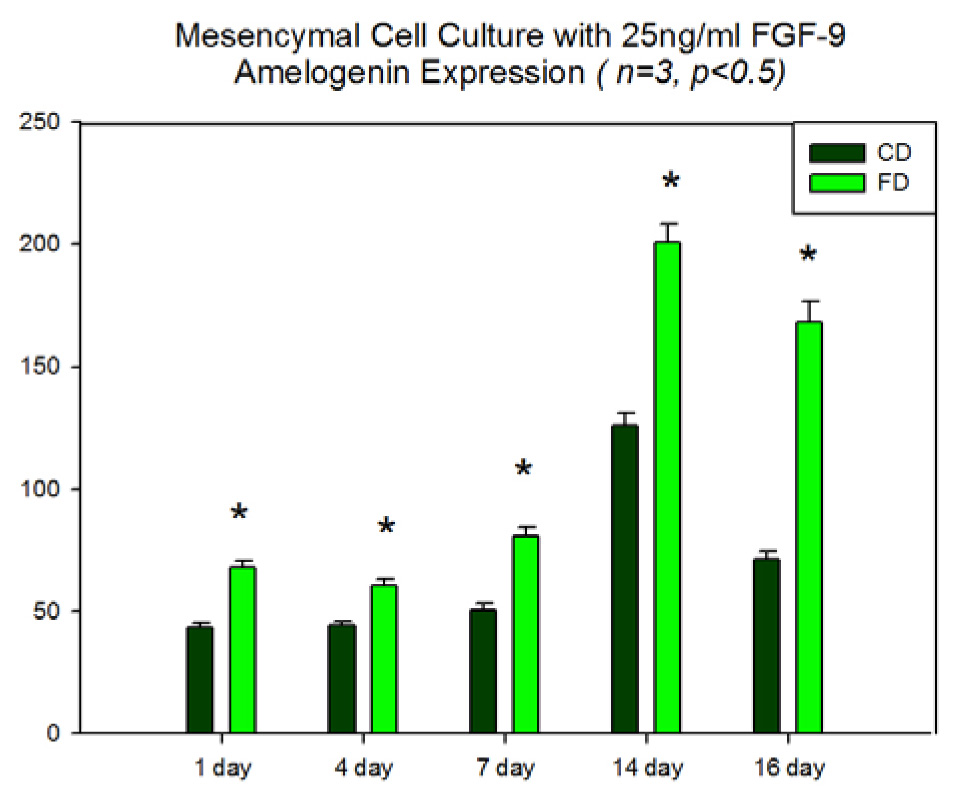


D E


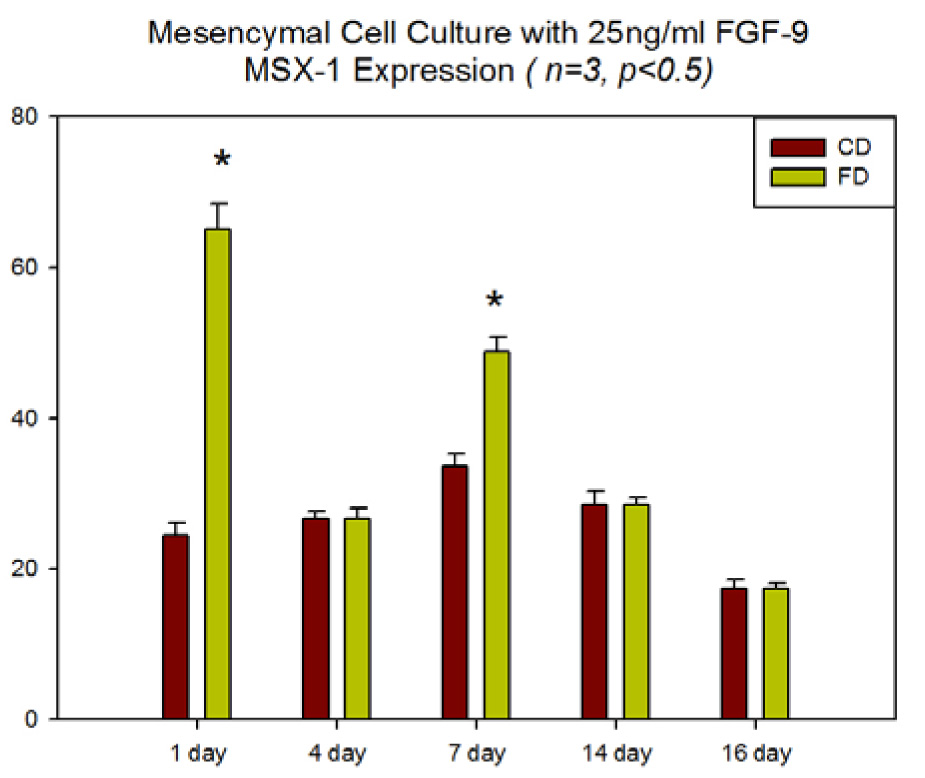

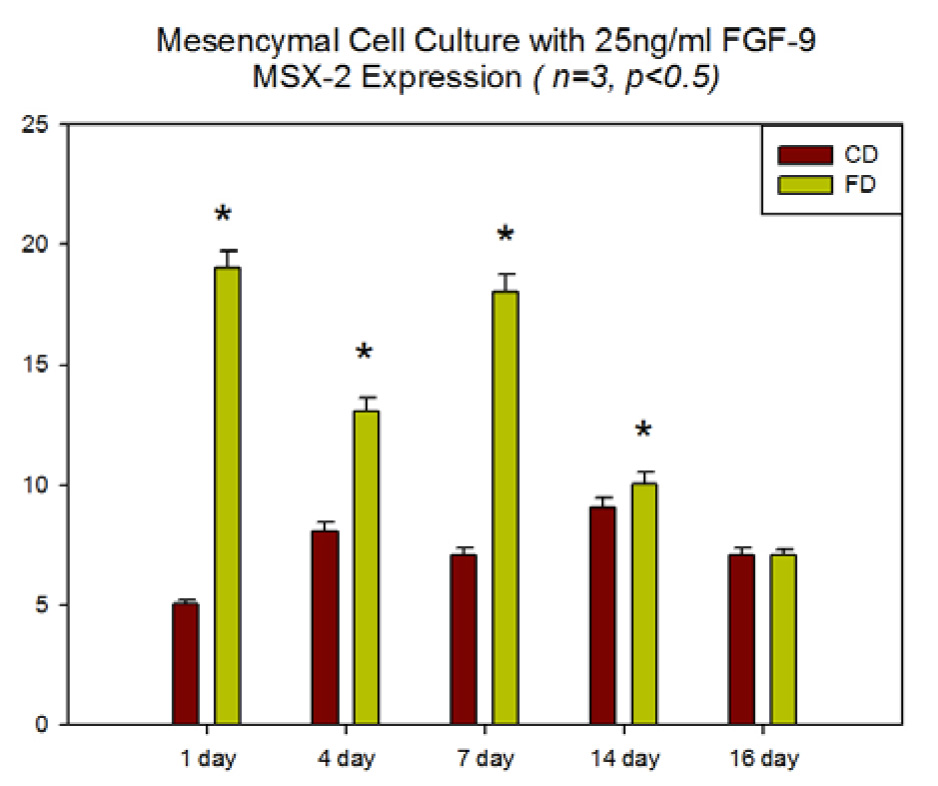


F G

**
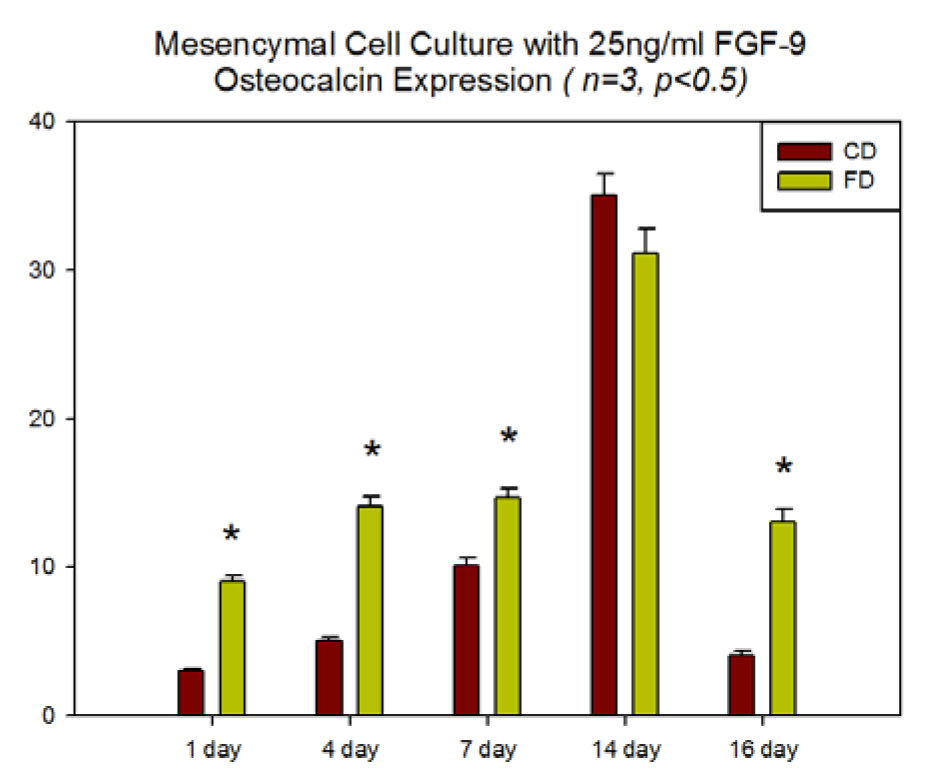

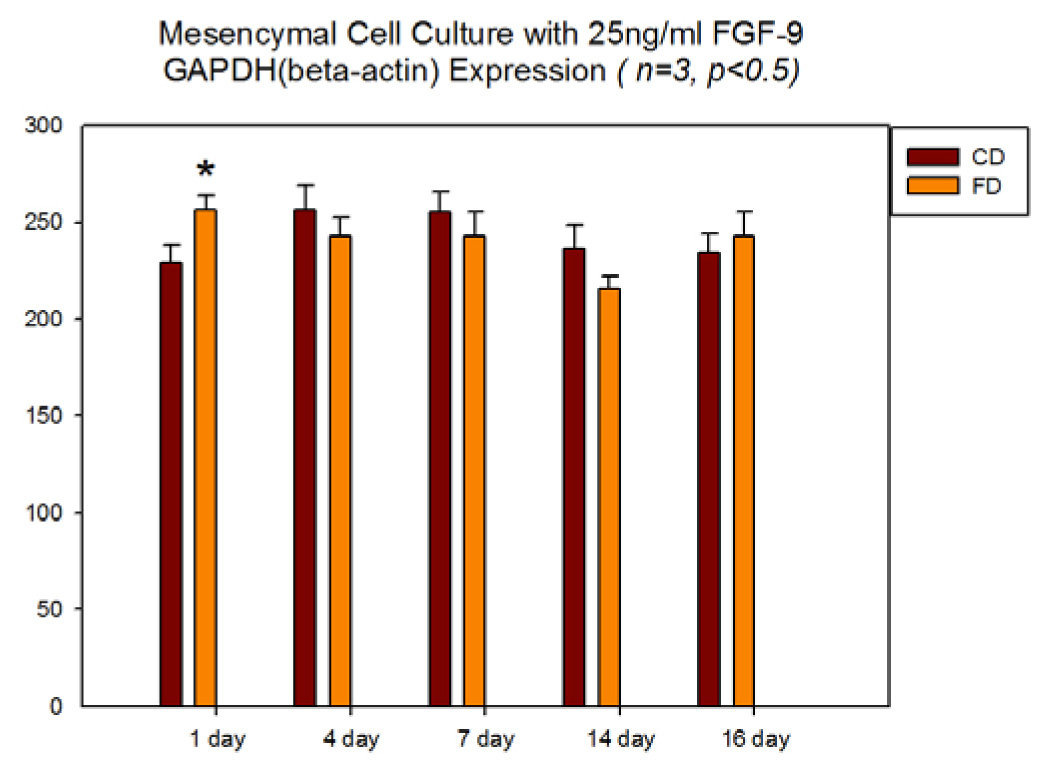
**

**Additional file 1.** Preliminary experiment: Fibroblast growth factor-9 (FGF-9) upregulates *Ameloblastin* and *Amelogenin* in cultured mesenchymal cells.

Mesenchymal cells were cultured *in vitro* and FGF-9 (25 ng/ml) was added to the experimental group. FGF-9 significantly upregulated *Ameloblastin* and *Amelogenin* expression. The homeobox protein *Msx genes Msx-1* and *Msx-2* (although expressed at low levels), and *Osteocalcin* were also upregulated after initial contact with FGF-9. *Msx-1* and *Msx-2* are critical to tooth germ development. Upregulation of *Msx-1* and weak expression of *Msx-2* evident in this study agree with previous research [19, 20]. *Osteocalcin* was upregulated by FGF-9 in the first week and high expression was sustained compared with the control group, only diminishing temporarily at the 14^th^ day. These results highlight the importance of FGF-9 in ectodermal organogenesis. **(A)** Electrophoresis of *Ameloblastin, Amelogenin*, *Msx-1*, *Msx-2*, *Osteocalcin,* and *β-actin* expression. We used *β-actin* as internal control. **(B)** *Ameloblastin* expression of mesenchymal cell cultured with FGF-9 *in vitro*, **(C)** *Amelogenin* expression, **(D)** *Msx-1* expression, **(E)** *Msx-2* expression, and **(F)** *Osteocalcin* expression. **(G)** Internal control of the preliminary experiment: *β-actin* expression. *(n = 3, p < 0.5) CD: control group. FD: FGF-9 group.*
