# Supplementary material for: FGF-9 accelerates epithelial invagination for ectodermal organogenesis in real time bioengineered organ manipulation
Source: Cell Commun Signal. 2012 Nov 23;10:34. doi: 10.1186/1478-811X-10-34 (PMC3515343; doi:10.1186/1478-811X-10-34)
Supplement: Additional file 2 — Haematoxylin and eosin staining of ectodermal organ culture. Day-1. (n=4) (A) and (B) control group Day 1. (C) and (D) FGF-9 group Day 1. Tooth germs cultured with FGF-9 demonstrated more differentiated than that in the control group. In this study, eight embryonic tooth germs were cultured, 4 for experimental group cultured with FGF-9, 4 for control group cultured without FGF-9. Some mineralized epithelium and morphological characteristics of ameloblasts were found in the experimental group with FGF-9, (C) and (D). (A) and (C): 200×; Bar = 12.5 μm. (B) and (D): 400×; Bar = 25 μm; 1: epithelium. 2: mesenchyme. [file 1478-811X-10-34-S2.doc]

**Additional file 2**

A B


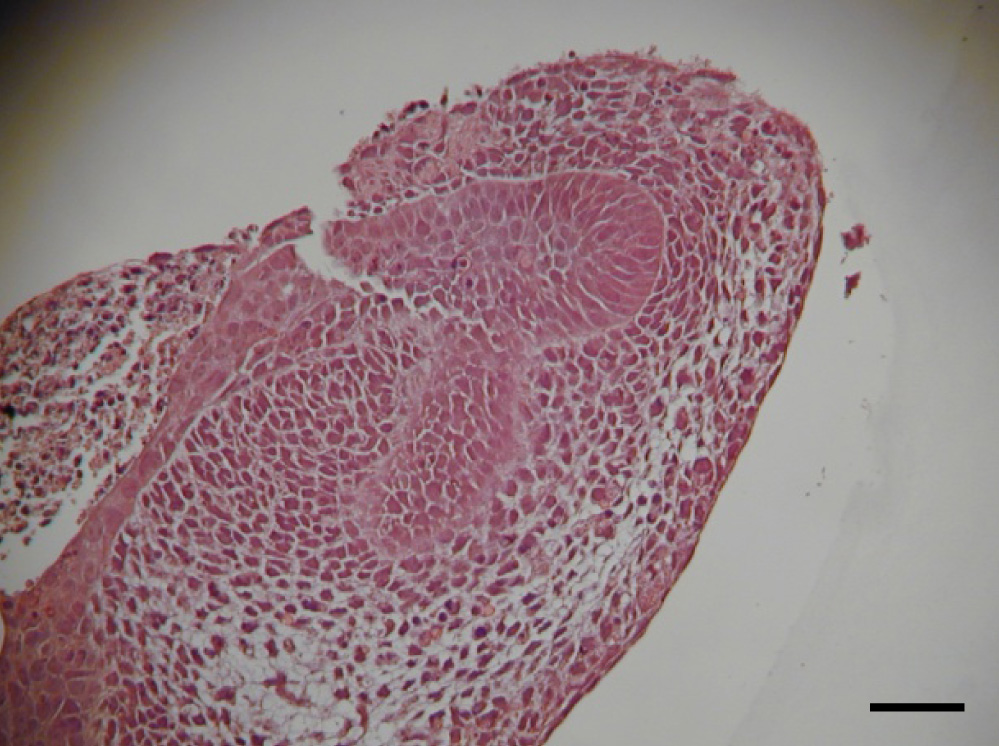

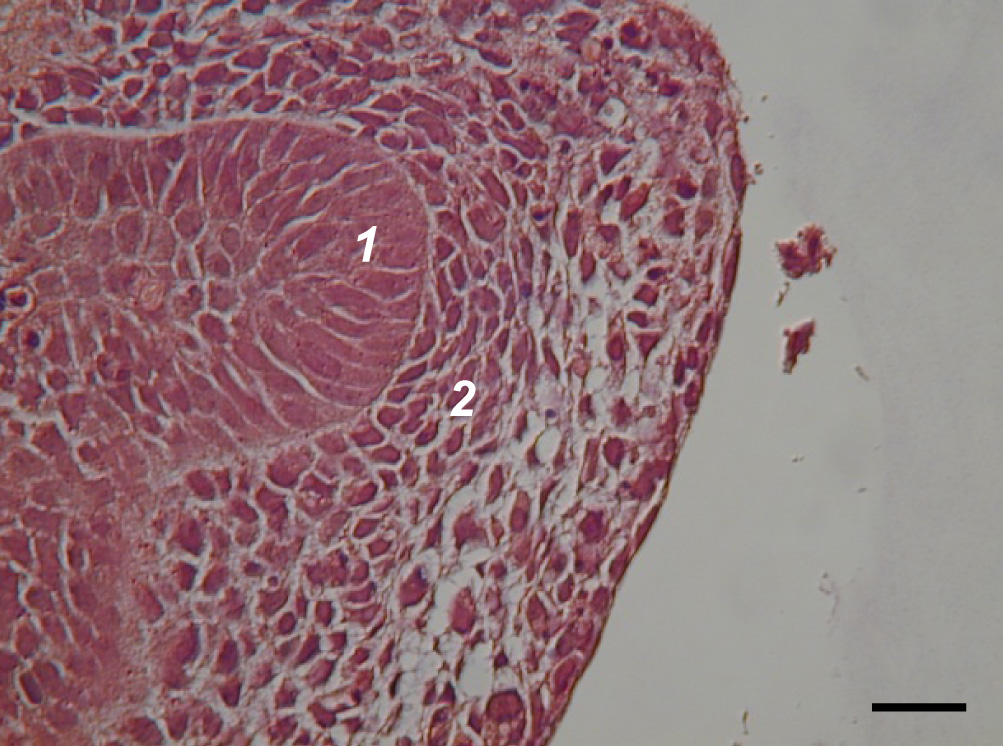


C D


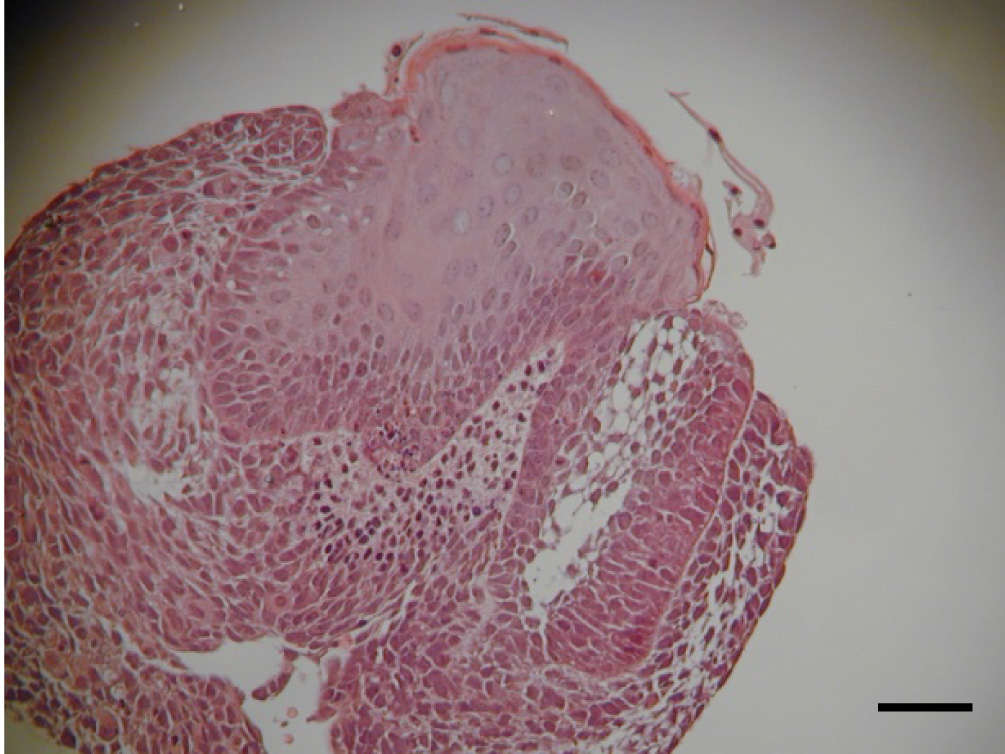

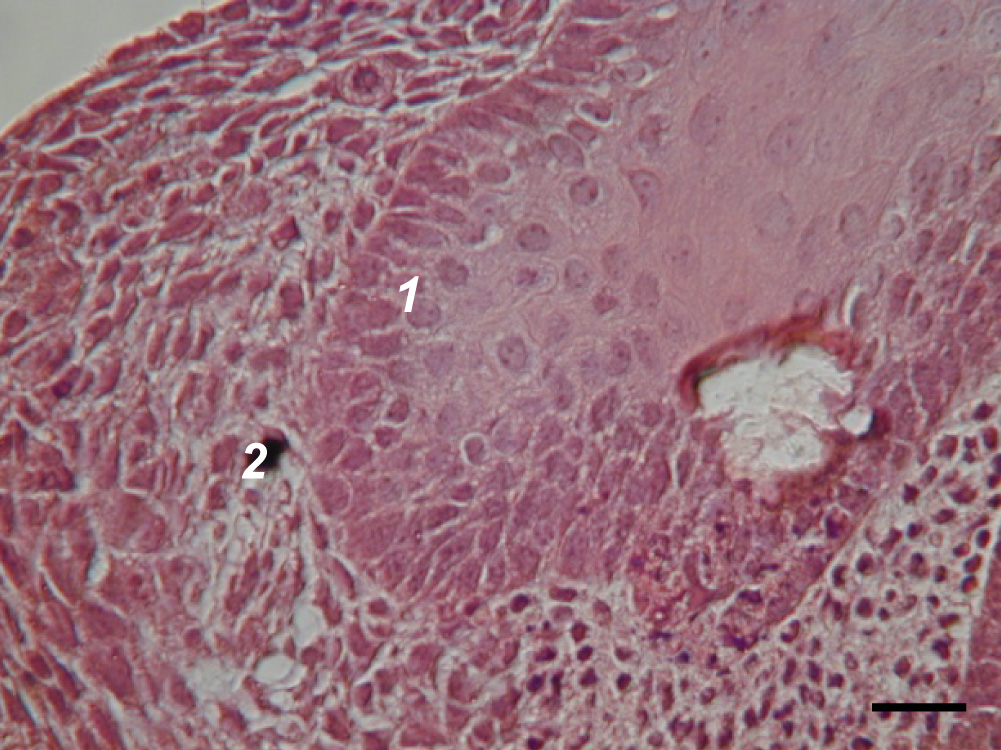


**Additional file 2.** Haematoxylin and eosin staining of ectodermal organ culture. Day-1. *(n=4)*

**(A)** and **(B)** control group Day 1. **(C)** and **(D)** FGF-9 group Day 1. Tooth germs cultured with FGF-9 demonstrated more differentiated than that in the control group. In this study, eight embryonic tooth germs were cultured, 4 for experimental group cultured with FGF-9, 4 for control group cultured without FGF-9. Some mineralized epithelium and morphological characteristics of ameloblasts were found in the experimental group with FGF-9, (C) and (D).

**(A)** and **(C)**: 200×; Bar = 12.5 μm. **(B)** and **(D)**: 400×; Bar = 25 μm ; *1: epithelium. 2: mesenchyme*.
