# Supplementary material for: FGF-9 accelerates epithelial invagination for ectodermal organogenesis in real time bioengineered organ manipulation
Source: Cell Commun Signal. 2012 Nov 23;10:34. doi: 10.1186/1478-811X-10-34 (PMC3515343; doi:10.1186/1478-811X-10-34)
Supplement: Additional file 3 — Haematoxyliin and eosin staining of ectodermal organ culture. Day 10. (n=4) Eight embryonic tooth germs were cultured, 4 for experimental group cultured with FGF-9, 4 for control group cultured without FGF-9. (A) control group Day 10. Only simple epithelium and mesenchyme in the tooth germ was found. 1: epithelium. 2: mesenchyme. (B) FGF-9 group Day 10. The FGF-9 group showed a more complex tooth germ structure after organ culture in vitro for 10 days. 3: some morphological characteristic of ameloblasts. 4: some morphological characteristic of odontoblasts. 5: mesenchyme. Bar = 25 μm. Magnification 400×. [file 1478-811X-10-34-S3.doc]

**Additional file 3**

A B


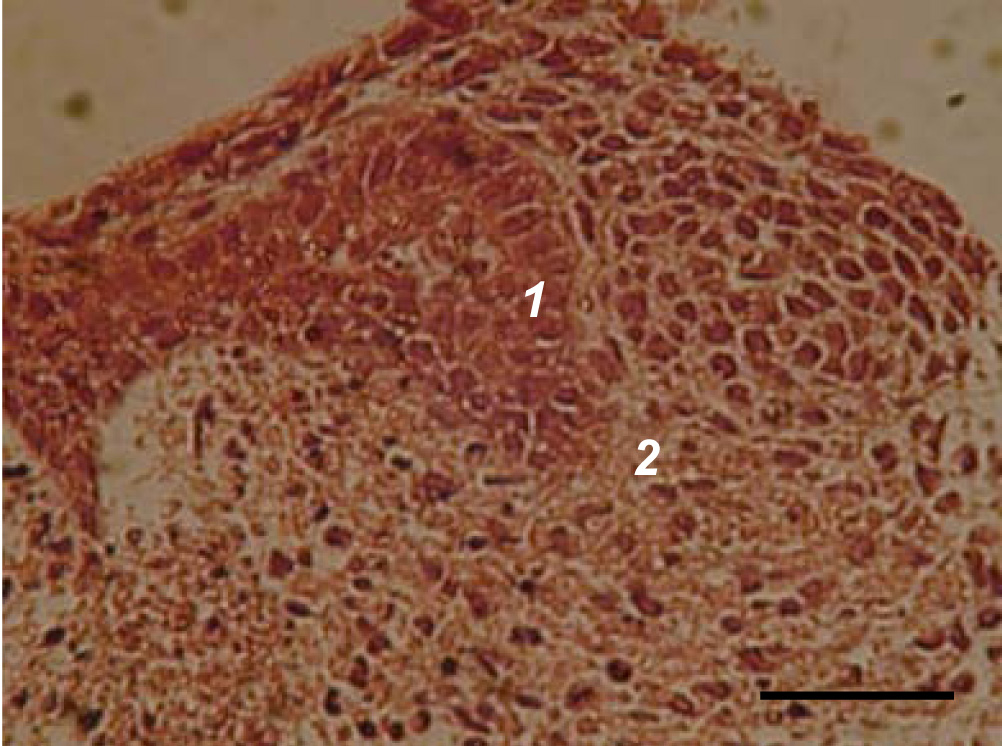

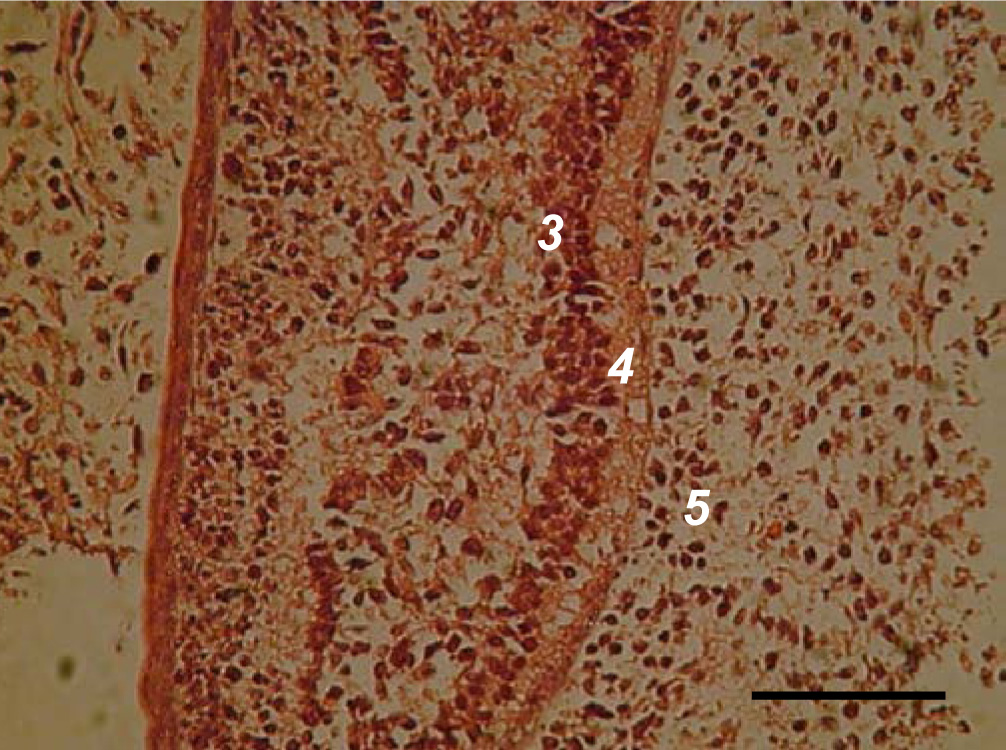


**Additional file 3.** Haematoxyliin and eosin staining of ectodermal organ culture. Day 10. *(n=4)*

Eight embryonic tooth germs were cultured, 4 for experimental group cultured with FGF-9, 4 for control group cultured without FGF-9.

**(A)** control group Day 10. Only simple epithelium and mesenchyme in the tooth germ was found. *1: epithelium. 2: mesenchyme.*

**(B)** FGF-9 group Day 10. The FGF-9 group showed a more complex tooth germ structure after organ culture *in vitro* for 10 days. *3: some morphological characteristic of ameloblasts. 4: some morphological characteristic of odontoblasts. 5: mesenchyme.* Bar = 25 μm. Magnification 400×.
